# From Dormancy to Viability: The Resuscitation Processes of Viable but Non-Culturable Bacteria—A Systematic Review

**DOI:** 10.3390/microorganisms14010136

**Published:** 2026-01-07

**Authors:** Prisca Tchato, Karine Marion-Sanchez, Talyssa Lebielle, Claude Olive

**Affiliations:** 1 Hygiene and Environment Laboratory, Department of Bacteriology, Centre Hospitalier Universitaire de Martinique, CS 90632, 97290 Fort-de-France, France; karine.sanchez@chu-martinique.fr (K.M.-S.);; 2 Department of Molecular Medicine, University of Siena, 53100 Siena, Italy; 3 Pathogenesis and Control of Chronic and Emerging Infections, University of Montpellier, INSERM, University of the Antilles, 97200 Fort-de-France, France

**Keywords:** systematic review, bacterial resuscitation, VBNC (viable but non-culturable), viable but non cultivable, bacterial dormancy, nosocomial infections, Enterobacteriaceae, *Pseudomonas aeruginosa*

## Abstract

Viable but non-culturable (VBNC) cells represent a reversible, metabolically active state that promotes the survival of bacteria under stressful conditions and their persistence in healthcare facilities and food industry. We conducted a systematic review following PRISMA 2020 guidelines to identify in vitro methodologies for inducing and resuscitating VBNC Enterobacteriaceae and *Pseudomonas aeruginosa*, and to determine key influencing factors. Eligible studies reported in vitro resuscitation of these species. Searches were performed in MEDLINE (PubMed), Scopus, and Google Scholar up to July 2025. Two independent reviewers screened and extracted data. Exclusion criteria included absence of original experimental data, focus on other species, or lack of clear VBNC definition. Risk of bias was qualitatively assessed. Analyses were descriptive without meta-analysis. Of the 1041 records, 24 articles (27 studies) were included. Resuscitation protocols typically employed standard culture media with additives and moderate incubation temperatures, with most successful recoveries occurring after 24–48 h. *P. aeruginosa* generally required less supplementation than Enterobacteriaceae. Reported mechanisms involved metabolic reactivation, oxidative stress modulation, nutrient sensing, and ribosome reactivation. The limitations of our study include protocol heterogeneity, lack of standardization, and selective reporting. While simple resuscitation methods were often effective, tailoring conditions to species-specific ecological preferences appears critical. Standardized approaches of VBNC cells will improve detection, risk assessment, and infection control.

## 1. Introduction

The ability of bacteria to enter a dormant state while remaining viable, referred to as the ‘viable but non-culturable (VBNC)’ state, constitutes a fundamental survival strategy in response to various environmental stresses, such as chemical disinfection, UV irradiation, dehydration [1], and nutrient limitation [2,3,4].

Concurrently, persistent cells (or “persisters”) represent a stress-tolerant subpopulation capable of withstanding hostile conditions without harbouring specific genetic mutations [5,6], whereas certain VBNC bacteria possess distinct mutations in genes such as rpoS, envZ, or fleQ [7,8,9], which regulate stress response pathways and facilitate survival under adverse conditions while preserving the ability to reactivate.

Studies by Yamasaki, Song, and Govers [6,10,11] suggest that the distinction between ‘persistent’ and ‘VBNC’ cells is largely a matter of terminology rather than a difference in fundamental biological characteristics. Persistence, dormancy, and the VBNC state are three bacterial survival strategies under stress, each modulating metabolic activity and growth recovery differently. They share common regulatory pathways, particularly those involving energy metabolism and oxidative stress. These three terms describe dormant, stress-adapted phenotypes capable of surviving and reactivating under appropriate conditions.

In both cases, these dormant states enable the pathogenic bacteria examined in this study to withstand antimicrobial interventions, persist in hospital or industrial environments, and re-emerge unpredictably when favourable conditions are restored [12,13,14]. These bacteria remain undetectable by conventional hospital environmental monitoring methods and their presence may substantially impact the transmission of healthcare-associated infections [15]. Based on the results of the National Survey on the Prevalence of Healthcare-Associated Infections [16], Enterobacteriaceae (notably *Klebsiella pneumonia*) and *Pseudomonas aeruginosa* are among the most frequently isolated multidrug-resistant organisms in healthcare-associated infections in the French West Indies. Their persistence in biofilms, combined with multidrug resistance traits, highlights their epidemiological importance and justifies the dual focus of this review.

The transition from dormancy to active growth relies on complex mechanisms that are still poorly characterized. This process involves the restoration of ribosomal activity and intracellular metabolism, as well as the detection and integration of specific environmental signals [10,11,17]. Despite growing interest in these processes, our understanding of them remains incomplete. Existing studies highlight significant heterogeneity, reflecting both the diversity of bacterial species examined and the wide range of experimental conditions employed [18,19,20].

Against this backdrop, the present systematic review aims to address the following question: which methodologies enable the resuscitation of VBNC bacteria and persister cells in vitro?

The primary objective of this work is to systematically analyze the methodologies described in the scientific literature with the aim of defining one or more resuscitation protocols applicable to two bacterial groups highly implicated in healthcare-associated infections: Enterobacteriaceae and *Pseudomonas aeruginosa*. We assume that these methods will be rather complex.

When data are available, we will also describe the molecular mechanisms involved in the resuscitation process.

Furthermore, this study aims to identify, based on the literature, the main conditions promoting the induction of the VBNC state and the main techniques for quantifying VBNC cells.

## 2. Material and Methods

This systematic literature review was conducted in accordance with the PRISMA 2020 (Preferred Reporting Items for Systematic Reviews and Meta-Analyses) guidelines.

The research question was as follows: what are the most effective current methods for the in vitro resuscitation of VBNC and which cellular mechanisms are involved in this process?

### 2.1. Eligibility Criteria

Eligible studies were original, peer-reviewed research articles published in English, French, Spanish, or Italian. To be included, the studies had to focus on Enterobacteriaceae or *Pseudomonas aeruginosa* and explicitly investigate the viable but non-culturable (VBNC) state. Studies were required to address at least one of the following aspects: the experimental methods and media used to resuscitate VBNC bacteria, the molecular, physiological, or environmental mechanisms involved in resuscitation, the induction or maintenance of the VBNC state under defined laboratory conditions, the characterization of bacterial dormancy at metabolic, structural, or morphological levels, or the quantitative approaches used to detect or enumerate VBNC cells. Articles were considered regardless of their geographic origin, publication year, or laboratory setting, provided that they reported primary experimental data. Both successful and unsuccessful resuscitation attempts were eligible, as long as the VBNC state was confirmed according to accepted criteria such as loss of culturability combined with maintenance of metabolic activity or membrane integrity.

Secondary publications such as reviews, systematic reviews, meta-analyses, commentaries, conference abstracts, or letters to the editor were excluded. We also excluded investigations lacking original experimental data, studies focusing on bacterial groups or species other that Enterobacteriaceae or *Pseudomonas aeruginosa* and reports that did not clearly define or confirm the VBNC state.

### 2.2. Information Sources and Search Strategy

An exhaustive literature search was conducted in two major databases, MEDLINE via PubMed and Scopus, and one search engine, Google Scholar. The search period covered publications up to and including July 2025, with no start date specified. Search equations combined the following keywords, individually or with Boolean operators (“AND”, “OR”): Bacterial resuscitation, VBNC (viable but non-culturable), viable but non-cultivable, bacterial dormancy, environmental stress, Enterobacteriaceae, *Pseudomonas aeruginosa*, and nosocomial infections. Search strategies were adapted for each database to optimize sensitivity and specificity.

### 2.3. Selection Process

All the references that were identified were imported into the Covidence platform.

Duplicates were removed automatically and verified manually. The selection process was conducted in two stages: first, the titles and abstracts of potentially relevant articles were screened; then, the full texts of these articles were reviewed.

Two reviewers performed each stage independently, and any discrepancies were resolved through discussion and consensus.

### 2.4. Data Collection Process

Data extraction was performed independently by two reviewers using a standardized form that we created. For each included study, we recorded the following information: bibliographic details (first author, year of publication, journal and country), the bacterial species investigated, and the study objectives. We extracted data regarding resuscitation methods, including the type of culture medium, the nature of supplementation, the incubation conditions, and the time required for recovery. When available, we documented quantification methods and environmental, physiological, or molecular factors associated with resuscitation. The experimental conditions used to induce the VBNC state were also noted. Articles with incomplete data were retained if they met the inclusion criteria.

### 2.5. Data Items

The primary outcomes of interest were resuscitation success (defined as the recovery of culturability), the time taken for recovery, and the cellular mechanisms involved. The secondary outcomes included the conditions of induction, confirmation of the VBNC state, and the quantification methods.

### 2.6. Risk of Bias Assessment

Due to the heterogeneity and in vitro methodological nature of the included studies, no formal, validated risk-of-bias tool was applied. However, potential sources of bias were qualitatively appraised, such as incomplete reporting of resuscitation criteria, non-standard confirmation of VBNC status and selective reporting of successful protocols.

### 2.7. Effect Measures

Given the descriptive nature and heterogeneity of the outcomes across the in vitro studies, no predefined quantitative effect measures were applied. Instead, outcomes were synthesized narratively.

### 2.8. Data Preparation

Qualitative descriptors (e.g., “rich” vs. “minimal” media) were harmonized by mapping them to reported compositions when available. Time units and temperature ranges were standardized. When outcomes were inconsistently defined (e.g., resuscitation success without culturability), a conservative interpretation was applied.

### 2.9. Synthesis Methods

The studies were grouped according to the type of bacterial groups (Enterobacteriaceae vs. *Pseudomonas aeruginosa*) and the resuscitation method used. The results were tabulated and displayed visually in structured tables and figures. A summary table was created and a narrative synthesis was performed to summarize the findings across the studies. Subgroup analyses explored fundamental differences between species and resuscitation approaches.

### 2.10. Reporting Bias Assessment

As no meta-analysis was undertaken, we did not perform formal tests for reporting bias (e.g., funnel plots). Potential selective reporting was considered qualitatively by noting the absence of protocols/registrations and inconsistencies between methods and reported outcomes.

### 2.11. Certainty of Evidence

Certainty of evidence was assessed qualitatively by considering the consistency of the results across the studies, the direct relevance of the in vitro outcomes to the review question, and the transparency of methods used to confirm the VBNC status and resuscitation. A formal grade assessment was not applicable due to the absence of pooled effect estimates.

## 3. Results

### 3.1. Study Selection

Among 1041 studies identified through our literature search, 167 duplicates were removed. After examining the titles and abstracts, 91 full-text articles were read in their entirety.

Ultimately, 24 articles met the eligibility criteria and were included in the analysis. Of the 24 articles, 3 dealt with both Enterobacteriaceae and *Pseudomonas aeruginosa*. This brings the total number of included studies to 27.

**Figure 1:**
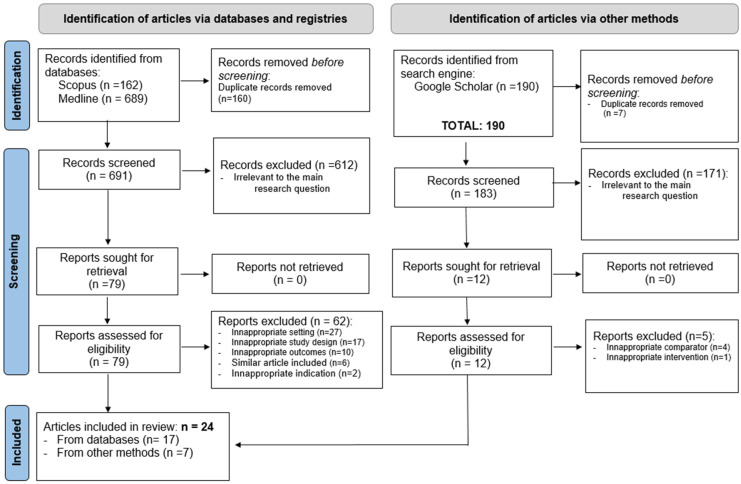
Prisma flow diagram.

The different stages of the article selection process are described in PRISMA flow diagram (Figure 1).

Of the ninety-one reports assessed for eligibility, sixty-seven were excluded for the following reasons: Twenty-seven studies were conducted in settings that did not align with the inclusion criteria. Seventeen studies employed designs that were methodologically incompatible with the scope of the review. Ten reports presented outcomes that were not relevant to the research question. Six articles were excluded due to thematic redundancy with articles that had already been included. Two reports addressed indications outside the scope of the review. Furthermore, five studies identified through supplementary methods were excluded due to inappropriate comparators (*n* = 4) or interventions (*n* = 1).

### 3.2. Study Characteristics

The main characteristics of the 24 articles included are summarized in Table 1.

All selected articles were published between 2003 and 2025, with a clear increase in the number of publications during the last decade. In fact, 66.6% (*n* = 16) of the articles analyzed were published after 2018.

The majority of the articles were from Asian and European countries, particularly China (33.3%, *n* = 8) and the United Kingdom (12.5%, *n* = 3). Other contributions originated from southern and western Europe, Africa (Egypt), and the United States, which contributed one article each.

Regarding publication outlets, the included articles were primarily published in journals covering environmental and microbiological sciences (41.5%, *n* = 8), such as Environmental Microbiology, Water Research, Applied and Environmental Microbiology, and Journal of Bacteriology.

In terms of bacterial models, Enterobacteriaceae, particularly *Escherichia coli*, were the most frequently studied organisms (58.3%, *n* = 14).

*Pseudomonas aeruginosa* was the second most investigated species (29.1%, *n* = 7), followed by *Klebsiella pneumonia* and *Proteus mirabilis*.

Three articles investigated both Enterobacteriaceae and *Pseudomonas aeruginosa*.

### 3.3. Primary Outcomes

Descriptive analysis of the mechanisms is given in Table 2.

### 3.4. VBNC Resuscitation Methods

In this subsection, percentages are calculated with reference to the 19 studies summarized in Table 1, which specifically report resuscitation-related data.

Respectively, 15 studies (78.9%) described VBNC cells resuscitation protocols applied to Enterobacteriaceae and 4 studies (21.1%) applied to *Pseudomonas aeruginosa*.

Of the 19 studies, 3 reported unsuccessful resuscitation [7,21,23]. In Boaretti’s research, the use of solid agar likely hindered the diffusion of extracellular growth factors necessary for resuscitation, thereby preventing the awakening of VBNC cells.

#### 3.4.1. Culture Media

All studies reported at least one type of medium used for resuscitation. Luria–Bertani (LB)-based formulations were the most frequently employed (36.8%, *n* = 7), appearing in liquid, solid, or agarose pad forms. Other studies employed alternative formulations such as tryptic soy broth (*n* = 2), M9–Glucose, Yeast–Peptone–Dextrose (YPD), or minimal salt–based media diluted with sea water or phosphate-buffered saline (PBS). Two studies employed the term “nutrient broth” without additional precision.

#### 3.4.2. Additives

Nutrient enrichment was a key strategy for 68.2% (*n* = 13) of the 19 studies. They included at least one additive to promote VBNC resuscitation. The most common supplements were catalase [21,32] and sodium pyruvate [7,25,27], both of which acting as antioxidants or reactive oxygen species scavengers. Additives such as amino acid mixtures were used by Pinto and Li, occasionally combined with proteorhodopsin activation under green light [32], to stimulate metabolic reactivation. Other chemical agents such as Tween 20 [26], sodium thioglycolate [27], or sodium diethyldithiocarbamate trihydrate (DDTC) served as surfactants or metal chelators to facilitate recovery.

Three studies [19,21,30] used cell culture supernatants or spent media to provide signalling molecules or metabolic by-products conducive to cell recovery. In contrast, four studies [14,23,29,34] performed resuscitation without any supplementation, relying solely on the nutrient content of the base medium.

### 3.5. Other Experimental Conditions

The incubation temperatures in all 19 studies ranged from 20 °C to 37 °C. The majority of the studies adopted moderate incubation conditions between 30 °C and 37 °C, corresponding to the optimal physiological range for the strains being tested. Lower temperatures (around 20 °C) were chosen in four studies [21,22,24,26]. Depending on the protocol, incubation was carried out either statically or under shaking conditions. In two cases [21,24], experiments were conducted in darkness to avoid photoactivation of pigments or photoreceptors. Some authors also incorporated stress modulation strategies, such as antibiotic protection [7,21,33], metal exposure [22,24], or phage challenge [33], to examine their effect on resuscitation capacity.

#### 3.5.1. Time to Recover

Resuscitation duration times were highly variable, ranging from 6 h [33] to 15 days [19]. Of the 16 studies that reported a resuscitation duration time, the majority (81.2%, *n* = 13) reported successful resuscitation within 24 to 48 h, usually under moderate temperature and nutrient-supplemented conditions.

The three unsuccessful cases [7,21,23] remained culture-negative despite extended incubation or supplementation attempts, highlighting the persistence of VBNC states under suboptimal conditions.

#### 3.5.2. Comparative Observations

Enterobacteriaceae generally responded more efficiently to nutrient-rich or antioxidant-supplemented media, with long resuscitation duration times (from 24 h to 15 days).

In contrast, *Pseudomonas aeruginosa* required a shorter resuscitation time of typically 24 h and often needed less nutrient availability to resume growth.

### 3.6. Cellular Mechanisms During the Resuscitation

A descriptive analysis of the mechanisms is provided in Table 3, which compiles data from eight studies and details the cellular mechanisms involved in resuscitation as well as the supporting evidence of successful resuscitation.

#### 3.6.1. Metabolic Reactivation

This group includes mechanisms that restore or enhance metabolic activity to support the revival of VBNC cells.

Li et al. [32] identified elevated intracellular ATP levels as a key mechanism during resuscitation in *Escherichia coli*. These elevated levels of ATP provide the energy required for cellular processes to restart, enabling VBNC cells to return to a culturable state.

Zhao et al. [33] indicated that ethanol production is restored in resuscitated *K. pneumoniae*, with the extent of restoration depending on environmental conditions and bacterial strains. This suggests that metabolic pathways, such as those involved in ethanol production, are reactivated during resuscitation, reflecting the recovery of metabolic functionality.

#### 3.6.2. Stress Response Modulation

The mechanisms in this group reduce environmental or cellular stress to facilitate resuscitation.

Wasfi’s data [26] emphasized the importance of temperature in resuscitation. The study showed that reaching an optimal temperature enhances catalase activity, allowing toxic peroxides to be degraded. This detoxification process is necessary for reviving VBNC cells of *Proteus mirabilis*, as it reduces oxidative stress that could otherwise bother cellular resuscitation.

#### 3.6.3. Nutrient Sensing and Signalling

This category encompasses the mechanisms involved in sensing environmental signals or nutrient availability in order to promote resuscitation.

Yamasaki’s research [6] identified alanine as a key signal for awakening persister cells in *Escherichia coli.* Six proteins associated with alanine metabolism (PsiF, PanD, YmgF, YjcF, PptA, and CheY) were found to enhance resuscitation. The study also described mechanisms such as reduced cyclic AMP (c-AMP) levels and activation of hibernating ribosomes to facilitate revival. In the context of VBNC cells, ribosomes often enter a “hibernating” state, where they are temporarily inactive in order to preserve energy under stressfull conditions. The activation of hibernating ribosomes is the process by which these ribosomes are reactivated to resume protein synthesis, which is essential for cellular resuscitation. This mechanism specifically involves releasing of ribosomes from their inactive state, which is often facilitated by environmental signals such as alanine. This enables the translation of proteins necessary for metabolic and cellular functions. This reactivation is a critical step in transitioning VBNC cells back to a culturable state, as it restores the cellular machinery needed for cellular growth and division.

Furthermore, resuscitation is mediated by chemotaxis sensors and phosphotransferase membrane proteins, which are likely to play a role in detecting environmental signals and initiating cellular resuscitation.

Song’s research [11] focused on the phosphate-sensing system as a driver of resuscitation in *Escherichia coli*. The transcriptional regulator PhoP activates PhoB through the small regulatory protein MgtS. Additionally, PsiF, a regulator associated with the phosphate response, and MgtA, a magnesium transporter regulated by the Pho regulon, were identified as important factors in enhancing resuscitation.

#### 3.6.4. Genomic and Phenotypic Changes

Pinto’s study [19] used csM13 minisatellite-PCR fingerprinting to confirm that resuscitated *Escherichia coli* cells were genomically identical to their original strains in all the tested media. This suggests that the resuscitation process does not introduce significant genetic alterations, meaning the revived bacteria have the same genetic profile as their pre-VBNC counterparts.

In contrast, Saima’s study [27] examined genomic changes that occur during the VBNC resuscitation cycle. By analyzing genomic distances between resuscitated isolates and their original strains, the study revealed alterations occurring during resuscitation. These findings suggest that while resuscitation restores viability, it may also induce subtle genomic changes that could potentially affect the characteristics of the revived *Escherichia coli*.

Yadav’s research [30] showed that resuscitated *K. pneumoniae* exhibited significantly higher resistance to common antibiotics than their original strains.

### 3.7. Secondary Outcomes

Descriptive analysis of the VBNC state in Enterobacteriaceae and *Pseudomonas aeruginosa* are given in Table 4 (including 19 studies) and Table 5 (including 8 studies).

#### 3.7.1. VBNC Induction: Culture Media, Additives, and Growth Conditions

For Enterobacteriaceae, a wide variety of culture media were used to induce the VBNC state. However, most studies (63.2%, *n* = 12) used nutrient-poor liquid environments (i.e., 0.9% NaCl, deionized water) to simulate starvation or osmotic stress.

However, nutrient-rich media (i.e., Luria–Bertani (LB) broth, Yeast Extract Tryptone broth 2XYT) were also used and additives (mainly antibiotics or disinfectants) were frequently employed to improve stress induction. Incubation temperatures ranged from −20 °C to 37 °C.

In contrast, *P. aeruginosa* studies predominantly utilized nutrient-poor media. Additives were less varied and primarily consisted of disinfectants or copper sulphate. Unlike Enterobacteriaceae studies, those on *P. aeruginosa* did not report the use of antibiotics or extreme pH to induce the VBNC state. Only 25% (*n* = 2) of studies used LB broth as nutrient-rich medium. Incubation temperatures were typically moderate (20–37 °C), with the exception of Zhao’s study which specified anaerobic conditions combined with a low temperature of 4 °C.

#### 3.7.2. Duration of VBNC Induction

The time required to induce the VBNC state in Enterobacteriaceae varied widely, ranging from 30 min (e.g., chlorination or antibiotic exposure) to 33 weeks (e.g., starvation in sterile distilled water). Short durations (30 min to 1 day) were associated with chemical disinfectants or antibiotics, while longer durations (14 to 33 weeks) were linked to starvation and low-temperature conditions.

For *P. aeruginosa*, induction times were generally shorter, ranging from 30 min to 7 days, with most studies reporting durations of 1 to 72 h. These shorter induction times were often associated with exposure to disinfectant (e.g., chlorine, UV, PAA).

#### 3.7.3. Confirmation of VBNC State

Of the 27 studies analyzed (19 for Enterobacteriaceae and 8 for *Pseudomonas aeruginosa*), 24 (88.89%) confirmed the VBNC state using at least one parameter of Pan’s definition. Specifically, 15 studies (55.56%) demonstrated non-culturability on standard media, 16 studies (59.26%) confirmed maintained metabolic or respiratory activity, 13 studies (48.15%) verified undamaged membrane integrity, and 6 studies (22.22%) reported abnormal morphology. These findings suggest that metabolic and respiratory activity were the most frequently assessed parameters, while abnormal morphology was the least, notably absent from studies on *Pseudomonas aeruginosa*.

#### 3.7.4. Culturability on Current Media

Culturability tests for Enterobacteriaceae were performed in 57.9% of the studies (*n* = 11), using current media. Ten studies reported no growth, confirming the VBNC state while one (Ni et al. [13]) did not specify the results of culturability tests.

For *P. aeruginosa*, culturability was assessed in 50% of studies (*n* = 4) with no growth observed in most cases, except for one study reporting residual growth after peracetic acid (PAA) treatment.

#### 3.7.5. Metabolic/Respiratory Activity

Of the studies assessed, 66.6% (18 out of 27 in Table 4 and Table 5) examined the metabolic activity of Enterobacteriaceae (*n* = 13) and *P. aeruginosa* (*n* = 5) in the VBNC state, using various techniques such as fluorescence, luminescence, chromatography (GC-MS), Raman spectroscopy, FISH, BacTiter-Glo, and DyeTox13-qPCR. These methods revealed either maintained or reduced metabolic activity, thereby confirming bacterial viability despite non-culturability. In some cases, there were physiological changes like suppressed DNA replication and increased oxidative stress (Li et al. [32]).

Fluorescence and luminescence techniques, as well as chromatography detect VBNC bacterial metabolic activity by assessing substrate utilization (e.g., glucose for ATP) and metabolite production (e.g., fatty acids). Fluorescence techniques (FISH, DyeTox13-qPCR) reveal cellular integrity or enzymatic activity, luminescence techniques (BacTiter-Glo) measure ATP production, and chromatography techniques (GC-MS, Raman) identify metabolites or chemical changes.

Together, these methods confirm bacterial viability and pathogenic potential, even under stressful conditions such as disinfection.

#### 3.7.6. Membrane Integrity

Membrane integrity was assessed in 66.6% of studies (*n* = 18) involving Enterobacteriaceae and *P. aeruginosa* studies from Table 4 and Table 5, primarily using the LIVE/DEAD BacLight kit as the reference method and flow cytometry as a complementary technique. The LIVE/DEAD kit uses SYTO 9 (green fluorescence) to stain intact membranes indicating viable cells, and propidium iodide (red fluorescence) to stain damaged membranes, signalling cell death or compromise.

Most studies reported intact or partially damaged membranes, confirming that both Enterobacteriaceae and *P. aeruginosa* maintain membrane integrity in the VBNC state.

#### 3.7.7. Abnormal Morphology

Abnormal morphology was reported in 36.8% (*n* = 7) of Enterobacteriaceae studies. Changes such as cell elongation, shortening, or spherical shapes were observed via electron microscopy (SEM, TEM), Gram staining, or the elongation method. Ni’s study did not report abnormal morphology. These morphological changes suggest that stress-induced adaptations occur in the VBNC state. In contrast, none of the *P. aeruginosa* studies, explicitly reported abnormal morphology.

#### 3.7.8. VBNC Cell Quantification

Quantification of VBNC cells in Enterobacteriaceae and *P. aeruginosa* was conducted in 70.4% (*n* = 19) of studies (27 studies from Table 4 and Table 5), employing methods such as flow cytometry, qPCR, PMA-ddPCR, PMA-qPCR, hemocytometry, time-lapse fluorescence microscopy (TLFM), TaqMan-based qPCR targeting the 16S rRNA gene, and DAPI staining with fluorescence microscopy. Specific formulas were reported, with one study [34] providing a shared formula for both species, while another [28] described a subtraction method for *P. aeruginosa*, enabling precise enumeration of VBNC cells across these bacterial groups.

To completement the detailed extraction tables presented above, we have provided a summary table below which outlines the main operating conditions available for studying VBNC bacteria (Table 6).

## 4. Discussion

The viable but non-culturable state observed across a wide range of bacterial species represents a compelling link between environmental and medical microbiology. The studies reviewed here confirm that entry into the VBNC state, as well as subsequent resuscitation, involve a complex interplay between environmental signals, cellular integrity, and metabolic regulatory mechanisms. Contemporary research aims not only to demonstrate the existence of VBNC cells but also to elucidate how bacteria enter and exit this state and, above all, how these processes can be manipulated experimentally in vitro to achieve their resuscitation. It should be noted, however, that research on the resuscitation of VBNC cells is still in its infancy, with most studies on this subject dating back fewer than 10 years.

The thorough analysis of these 24 articles has enabled us to identify numerous techniques that could successfully resuscitate VBNC cells. Surprisingly, most of these methods were not overly complex. They mainly consisted of a standard culture medium that was enriched to varying degrees, sometimes in combination with a modified physical parameter. Methods to resuscitate VBNC *Pseudomonas aeruginosa* were even simpler, achieving results in as little as 24 h.

These observations suggest that potentially less complex techniques could potentially play a key role, prompting us to reconsider the degree of complexity of the methods suggested in our initial hypothesis. According to Stover’s study [35], the complete genome sequence of *P. aeruginosa* PAO1—the largest bacterial genome sequenced to date, at 6.3 million base pairs—revealed an extensive repertoire of regulatory genes, stress resistance systems, and metabolic pathways that confer remarkable adaptability to nutrient-limited environments [37]. This genetic versatility is likely to be the basis of its ability to recover more efficiently from the VBNC state than Enterobacteriaceae, which generally require richer nutrient availability.

It is important to emphasize that in order to allow resuscitation, cells should not be “overfed” with overly rich media. An excess of nutrients can paradoxically prevent VBNC cells from awakening by disturbing the subtle physiological signals necessary for resuming growth [38]. The inhibitory effect of overly rich media may be explained by the induction of oxidative stress, since excessive metabolic activity leads to the accumulation of reactive oxygen species. Additionally, complex nutrient environments can interfere with signalling pathways essential for VBNC exit, such as those involved in nutrient sensing or ribosomal reactivation. These mechanisms highlight the importance of balanced nutrient conditions, where moderate supplementation supports recovery without overwhelming cellular regulatory systems [3,33].

Choosing the most appropriate technique for resuscitating Enterobacteriaceae involves defining a set of criteria: (i) a culture medium enabling recovery with minimal complexity; (ii) a short resuscitation time; (iii) confirmation of viable yet non-proliferating cells before resuscitation time is initiated (including at least one parameter, supported by Pan et al. [39]).

For example, Boaretti’s study [7] in liquid phase, as well as Kim’s [14] and Zhu’s [29] studies, retained our attention because all three meet the above-defined criteria. In fact, Luria–Bertani broth, whether supplemented with sodium pyruvate or agarose pads or not, represents very simple growth conditions that leads to resuscitation within a very short time. Furthermore, the VBNC status is fully validated in all three studies. In our opinion, these approaches could represent a practical and accessible method for our future in vitro resuscitation experiments.

On the other hand, the complexity of Pinto’s study [19] also caught our attention, due to the extensive optimization of the media, additives, and conditions required for successful bacterial resuscitation.

This thorough screening ultimately concluded that simple techniques could be sufficient for VBNC resuscitation. In fact, the simplest method for resuscitating *E. coli* VBNC cells, as demonstrated in Pinto’s study [19], is to use TSB (Tryptone Soya Broth) at 37 °C, which effectively revives suspensions induced bu 4 °C without the need for complex additives.

In general, we have observed that resuscitation is most effective when bacteria are reintroduced to environmental conditions that mimic their natural habitats. For example, *P. aeruginosa*, which is commonly found in aquatic environments, regains viability when exposed to water or minimal nutrient conditions that resemble its ecological niche [22,32,34]. Conversely, Enterobacteriaceae, which typically thrive in the nutrient-rich intestinal environment, appear to require slightly richer growth media [19,26,32]. This is also described in Ziying Lu’s study [40] which found that *Salmonella enterica* requires a balanced, intestine-like medium to facilitate recovery from the VBNC state. Tailoring resuscitation conditions to the specific ecological preferences of each bacterial species thus seems critical for successfully restoring their metabolic activity and growth [41]. These observations highlight the need for a delicate balance between nutrient supply and gradual stimulation to optimize resuscitation.

The transition to the VBNC state is induced by various stressors that are often associated with disinfection processes or other growth-limiting conditions. These stimuli usually result in a significant decrease in cell size and metabolic activity, as well as triggering oxidative stress protection mechanisms. These adaptations enable cells to survive conditions that would be lethal for culturable populations. This resilience suggests that both Enterobacteriaceae and *P. aeruginosa* have the potential to enter the VBNC state as a protective mechanism in response to various stressors.

Enterobacteriaceae exhibit greater versatility in VBNC induction than *P. aeruginosa*. They respond to a broader range of stressors (e.g., antibiotics, extreme pH, high osmotic pressure) and media types, whereas *P. aeruginosa* is primarily induced by disinfectants and nutrient starvation. The shorter induction times for *P. aeruginosa* (30 min to 7 days) compared to Enterobacteriaceae (30 min to 33 weeks) suggest that it may more rapidly respond to stress, particularly to chemical disinfectants.

Numerous VBNC detection and quantification approaches are described in our study. Guo et al. [42] mention most of them, describing and evaluating them while pointing out their limitations. The metabolic activity-based method uses the uptake of dyes such as fluorescein diacetate (FDA) to detect viable bacteria by converting them into fluorescent signals via active enzymes. However, limitations such as the quenching effect and pH sensitivity should be noted. The ATP assay measures bioluminescence linked to ATP and is useful for rapid detection, but it is subject to interference from other ATP sources. Methods based on membrane integrity, such as dye exclusion assays (e.g., trypan blue, propidium iodide) and nucleic acid-based techniques (e.g., PMA-qPCR), identify VBNC bacteria via membrane selectivity, though they are limited by an inability to distinguish bacterial species. These methods enable the VBNC fraction to be quantified more accurately and allow predictive models of microbiological risk to be refined [13].

In real life, the conditions favouring VBNC induction are therefore multiple and interdependent. Beyond the chemical and physical stresses already mentioned, biofilm formation [28,42] provides an environment conducive to establishing a microclimate that is not favourable for growth but is favourable for prolonged survival. Nutrient and oxygen gradients within biofilms support differentiated metabolic states, including VBNC cells, which are difficult or even impossible to detect using conventional culture methods. In aqueous environments, such metabolic plasticity challenges the reliability of using *E. coli* as the sole indicator of contamination, emphasizing the need for complementary physiologically based detection methods [13,27].

Bacterial biofilms have recently been found in dry environments, particularly on dry surfaces in healthcare facilities [31,43]. According to Schapira et al. [44], these dry surface biofilms (DSBs) harbour VBNC bacteria, enhancing their persistence and making them harder to detect, which consequently raises concerns for infection control [44].

These observations lead us to reconsider VBNC state not as static, but as part of a physiological continuum between persistence, dormancy, and cell death.

This systematic review has several limitations that should be acknowledged.

First, VBNC resuscitation is a relatively recent topic, with most studies having been published within the last decade. Consequently, the available evidence is still limited in scope and heterogenenous, which restricts the strength of the conclusions that can be drawn. Second, the methodological variability between studies (including differences in bacterial strains, induction conditions, and resuscitation protocols) makes direct comparisons and synthesis of the findings difficult. Third, most studies were conducted under controlled in vitro conditions, which may not fully reflect the complexity of natural or clinical environments, resulting in interpretative limitations. This explains why this study could not be subjected to a meta-analysis. Consequently, the results should be interpreted as a qualitative synthesis. Future research should aim to harmonize methodologies, expand the range of bacterial species studied, and generate data that can be used in meta-analyses.

## 5. Conclusions

The VBNC state, which is observed in bacteria such as Enterobacteriaceae and *Pseudomonas aeruginosa*, is a resilient survival strategy in response to stressors such as disinfectants, nutrient scarcity, or desiccation. Considering VBNC as part of a continuum with persistence and dormancy highlights shared regulatory pathways and provides valuable insights into bacterial survival. Our systematic review reveals that simple resuscitation methods in liquid phase—such as Luria–Bertani broth or Tryptone Soya Broth—used under conditions tailored to the original bacterial ecological niches, can effectively restore viability. This challenges the assumption that complex approaches are necessary. Although modern detection methods such as DyeTox13-qPCR or PMA-qPCR improve VBNC quantification, limitations persist. The hiding of bacteria as VBNC inside DSBs in healthcare facilities greatly complicates their detection, as conventional methods based on culturability are unsuitable. Research is already in progress to explore these mechanisms in these high-risk ecosystems, with the aim of developing targeted interventions to enhance environmental monitoring and infection control.

## Figures and Tables

**Table 1:** Study characteristics.

	GENERAL INFORMATIONS				INCLUSION CRITERIA (Key Words)		AIM OF THE STUDY	
Article Number	First Author	Year	Countries	Journal	Primary Outcome	Secondary Outcomes	Enterobacteriaceae	*Pseudomonas aeruginosa*
**1**	Boaretti [7]	2003	Italy	Environmental microbiology	VBNC; Resuscitation	VBNC entry; Membrane integrity	To determine the role of rpoS and its regulator (p)ppGpp in controlling entry, persistence, and resuscitation of VBNC ***E. coli***	
**2**	Arana [21]	2007	Spain	FEMS Microbiology Ecology	Resuscitate; VBNC	Environmental factors;	To determine if VBNC ***E. coli*** cells can regain culturability when exposed to different media and stimuli	
**3**	Pinto [19]	2011	Portugal	Journal of Applied Microbiology	VBNC; Resuscitation	Strain-specific responses; Temperature change	To identify conditions that induce VBNC state in ***E. coli*** and assess resuscitation strategies	
**4**	Dwidjosiswojo [22]	2011	Germany	International Journal of Hygiene and Environmental Health	VBNC; Recovery	Membrane integrity; Cytotoxicity; Induction		To evaluate copper’s impact on ***P. aeruginosa*** viability and VBNC induction in drinking water conditions
**5**	Dolezalova [23]	2015	Czech Republic	Bioelectrochemistry	VBNC	Lipid peroxidation, Membrane permeability; DNA leakage; induction	To investigate the effects of atmospheric pressure plasma jet treatment on ***E. coli***, focusing on membrane damage, loss of culturability, and induction of a VBNC state	
**6**	Govers [10]	2017	England	Environmental Microbiology	Resuscitation	Stress; Protein aggregation;	To investigate intracellular protein aggregation dynamics to understand inactivation and resuscitation heterogeneity in stressed ***E. coli*** populations	
**7**	Dopp [24]	2017	Germany	International Journal of Medical Microbiology	VBNC	Membrane disruption; Mitochondrial activity		To investigate whether copper-induced VBNC ***P. aeruginosa*** retain virulence and cytotoxic effects on bronchial epithelial cells
**8**	Chen [2]	2018	China	Water Research	VBNC; Resuscitation	Cell integrity partially maintained; Induction	To investigate induction of VBNC ***E. coli*** by chlorination/chloramination, characterize physiological/biochemical traits, and test resuscitation	
**9**	Kim [14]	2018	USA	Environmental Microbiology	VBNC; Dormancy; Resuscitation	Starvation-induced persisters; Membrane damage;	To determine whether VBNC ***E. coli*** and persister states are distinct or identical stress responses	
**10**	Ogane [25]	2019	Japan	Biocontrol Science	VBNC; Resuscitation	Pyruvate; Food preservative; Induction	To investigate the effect of low concentrations of food preservatives/disinfectants on the induction of VBNC ***E. coli*** and assess resuscitation with sodium pyruvate	
**11**	Yamasaki [6]	2021	USA	iScience	Persister cell; Resuscitation	Membrane receptor signaling	To investigate mechanisms of persister cell resuscitation in ***E. coli***	
**12**	Wasfi [26]	2020	Egypt	Journal of Cellular and Molecular Medicine	VBNC; Resuscitation	Stress condition; Virulence and stress response genes; Induction	To characterize the induction and resuscitation of the VBNC state in ***P. mirabilis***	
**13**	Song [11]	2020	South Korea, USA, Japan	Environmental Microbiology	Persister; Resuscitation	Absence of nutrients; Persister formation	To assess how cryptic prophages influence persister cell resuscitation in ***E. coli***, particularly in response to nutrient signals	
**14**	Saima [27]	2021	Russia	Microbiology (Russian Federation)	VBNC; Resuscitation	Environmental stressors	To evaluate survival of ***E. Coli*** in water microcosms and reconsider its reliability as an indicator organism	
**15**	Wilks [28]	2021	UK	mBio	VBNC; Resuscitation	Biofilm formation; Induction	To investigate biofilm formation on urinary catheters and the induction of VBNC ***E. coli*** and ***P. mirabilis***	To investigate biofilm formation on urinary catheters and the induction of VBNC bacteria
**16**	Zhu [29]	2022	China; UK	Journal of Hazardous Materials	VBNC; Resuscitate	Disinfection strategy	To explore how chlorination and UV disinfection induce VBNC in ***E. coli*** and asses whether VBNC cells remain pathogenic or can resuscitate	
**17**	Yadav [30]	2022	India	Frontiers in Microbiology	VBNC; Resuscitation	Antimicrobial resistance post-resuscitation; Virulence genes; Membrane integrity; Formic acid Induction	To assess formic acid induced VBNC state and resuscitation effects on virulence and antimicrobial resistance traits in ***K. pneumoniae***	
**18**	Hu [31]	2015	China	Environmental Research	VBNC;	VBNC detection; Altered metabolism; Metabolic markers; chlorine stress; Induction		To characterize metabolic features and identify metabolic markers of VBNC ***P. aeruginosa*** induced by chlorine stress
**19**	Chiang [15]	2022	Singapore	Journal of Applied Microbiology	Loss of culturability; Dormant cells	Viability PCR; Disinfection	To investigate the physiological responses of nosocomial bacteria (***E. coli***, *P. aeruginosa*, *E. faecalis*, *Bacillus sphaericus*) to UV and chlorine disinfection	To investigate the physiological responses of nosocomial bacteria (*E. coli*, ***P. aeruginosa***, *E. faecalis*, *Bacillus sphaericus*) to UV and chlorine disinfection
**20**	Guo [3]	2018	China	Science of The Total Environment	VBNC	Biofilm; Disinfection		To investigate how chlorine disinfection induces bacteria in biofilms into a VBNC state and monitor structural changes using Optical Coherence Tomography
**21**	Li [32]	2024	China	Journal of Bacteriology	VBNC; Persister; Resuscitation	Intracellular ATP; Induction	To investigate how intracellular ATP concentration regulates bacterial cell fate, particularly the induction and resuscitation of VBNC and persister states in ***E. coli***	
**22**	Zhao [33]	2024	China	Applied and Environmental Microbiology	VBNC; Resuscitation	Survival strategies, Metabolic shifts; VBNC formation	To study the factors triggering VBNC state fomation and resuscitation in alcohol-producing ***K. pneumoniae***	
**23**	Fan [34]	2024	China	Water (Switzerland)	VBNC	Disinfection; Induction; Detection		To investigate the induction and detection of the VBNC state in ***P. aeruginosa*** during drinking water disinfection processes
**24**	Ni [13]	2025	China	Environmental Pollution	VBNC	q PCR detection	To assess occurrence, develop predictive models, and evaluate health risks of VBNC ***E. coli*** and *P. aeruginosa* in drinking water systems	To assess occurrence, develop predictive models, and evaluate health risks of VBNC *E. coli* and ***P. aeruginosa*** in drinking water systems

**Table 2:** Primary outcomes—VBNC resuscitation methods.

Article Nb	Enterobacteriaceae				*Pseudomonas aeruginosa*			
	Culture Media	Additives	Other	Time to Recover	Culture Media	Additives	Other	Time to Recover
**1. Boaretti** [7]	1. Solid media (LBA); 2. Liquid media (LB *)	Sodium pyruvate	Incubation at 30 °C; Benzopenicillin for 2 h in one experiment;	1. No resuscitation, as agar in the solid medium impeded diffusion 2. 4 days				
**2. Arana** [21]	Medium broth + 0.5% glucose + 0.03% yeast extract (MBGYE); Sterile sea water	1. Catalase; 2. Supernatants from growth cultures; 3. DLB (Diluent LB); 4. DLB + catalase	Incubation with shaking at 20 or 37 °C in darkness. Ciprofloxacin (0.08 μg/mL) and/or streptomycin (64 μg/mL); both antibiotics added to prevent residual culturable growth	≥6 days Unsuccessful resuscitation				
**3. Pinto** [19]	Screening on various culture media based on TSB or M9 at various dilutions	1. Amino acids; 2. Supernatants in TSB or M9 at various dilutions; 3. ATCC trace mineral supplement	Incubation at 25 or 30 or 37 °C	24 h to 15 days				
**4. Dwidjosiswojo** [22]					Deionized water	DDTC (sodium diethyldithiocarbamate trihydrate); Copper sulfate as control	Incubation at 20 °C	24 h
**5. Dolezavola** [23]	Luria-Bertani broth	None	-	Unsuccessful resuscitation				
**6. Govers** [10]	Agarose pads	LB + Ampicillin	Incubation at 37 °C	8 h				
**7. Dopp** [24]					Deionized water	Copper sulfate 10 µM + chelator sodium diethyldithiocarbamate trihydrate (DDTC)	Incubation at 20 °C in the dark	24 h
**8. Chen** [2]	Sterile LB broth	Sodium thiosulfate pentahydrate (0.12%, 1:100) to quench chlorine and chloramine	Incubation at 37 °C with shaking	32 h				
**9. Kim** [14]	Agarose gel pads	None	Incubation at 37 °C	24 h	Agarose gel pads	None	Incubation at 37 °C	24 h
**10. Ogane** [25]	Nutrient broth	Sodium pyruvate	Incubation at 37 °C	24 h				
**12. Wasfi** [26]	1. Nutrient broth 2. TSB (Tryptic Soy Broth)	1. None 2. Tween 20 (3% *v*/*v*) + low-nutrient medium Brain Heart Infusion BHI (1/8 concentration)	1. Incubation at 20 °C to 35 °C 2. Incubation 37 °C	1. 7 h 2. 72 h				
**13. Song** [11]	Agarose gel pads	M9 –Glu medium	Not specified	Not specified				
**14. Saima** [27]	1. Fresh TSB 2. LB broth 3. Peptone water 4. TSB medium	1. None 2. None 3. 0.5% sodium thioglycolate 4. 0.1% sodium pyruvate	Incubation at 37 °C	24 h to 48 h				
**16. Zhu** [29]	LB broth	None	Incubation at 37 °C	24 h				
**17. Yadav** [30]	Fresh media	1. Spent media (residuals of initial culture media); 2. Spent media + fresh media	Incubation at 37 °C	48 h				
**21. Li** [32]	PBS (nutrient-poor) and/or LB agar plates	Catalase + amino acids + proteorhodopsin + green light illumination + all-trans retinal	Incubation at 37 °C; With green light (5 min), without green light (35 min)	Not specified				
**22. Zhao** [33]	Fresh Yeast-Peptone-Dextrose YPD (1:100, vol/vol)	None	1. Incubation at 37 °C with shaking; 2. Incubation at 37 °C, antibiotics removed with PBS; Bacteriophage phiW14 (to assess their effects on resuscitation); Imipenem, ciprofloxacin, polymyxin added to prevent residual culturable growth	1. 6 h 2. Overnight				
**23. Fan** [34]					1. LB medium 2. Nutrient agar medium	None	1. Incubation at 37 °C 2. Incubation at 37 °C	1. 16 h 2. 24 h

* Luria-Bertani (LB) broth composition: 10 g tryptone, 10 g NaCl, 5 g yeast extract and 950 mL water.

**Table 3:** Cellular mechanisms during the resuscitation process—Enterobacteriaceae.

Article Nb	Cellular Mechanisms Involved in Resuscitation	Evidence of Resuscitation
**3. Pinto** [19]		csM13 minisatellite-PCR fingerprinting confirmed that cells resuscitated in all tested media were genomically identical to the original strains
**11. Yamasaki** [6]	Alanine is a true signal for waking persister cells. Six proteins from the alanine, were identified that increased waking: PsiF, PanD, YmgF, YjcF, PptA, and CheY. Reducing c-AMP levels and activating hibernating ribosomes. Resuscitation happens through the chemotaxis sensors and phosphotransferase membrane proteins.	
**12. Wasfi** [26]	Reaching the optimal temperature enhances catalase activity, allowing the degradation of toxic peroxides	
**13. Song** [11]	Activation of the phosphate-sensing system enhances resuscitation through PhoP (the transcriptional regulator), which activates PhoB via the small regulatory protein MgtS, as well as through PsiF, a regulator associated with the phosphate response, and MgtA, a Pho regulon-controlled magnesium transporter	
**14. Saima** [27]		Genomic distances between resuscitated isolates and their originals reveal changes occurring during the VBNC resuscitation cycle
**17. Yadav** [30]		Resuscitated bacteria showed significantly higher resistance to common antibiotics than original strains
**21. Li** [32]	Intracellular ATP elevation	
**22. Zhao** [33]	Ethanol production is restored, depending on environmental conditions and bacterial strains;	

All described mechanism can be regrouped in four categories.

**Table 4:** Secondary outcomes—Enterobacteriaceae.

Article Nb	Entry into VBNC State			VBNC State Confirmation (According to Pan’s Definition) Result/Method				VBNC Cells Quantification
	Culture Media +/− Additives	Growth Conditions	Duration Time	Culturability on Current Media	Metabolic/ Respiratory Activity	Membrane Integrity	Abnormal Morphology	Method
**1. Boaretti** [8]	Artificial Oligotrophic Medium (AOM) per liter: 500 mg NH_4_Cl + 100 mg NH_4_NO_3_ + 200 mg Na_2_SO_4_ + 300 mg K_2_HPO_4_ + 100 mg KH_2_PO_4_ + 10 mg MgSO_4_ + 8 g NaCl	Nutrient depletion through the culture media; Incubation at 4 °C.	From 21 to 33 days	No media specified No culture	CTC (5-cyano-2,3-ditolyl tetrazolium chloride) redox dye staining for the respiration activity	LIVE/DEAD BacLight kit (SYTO 9 + Propidium Iodide PI): green if intact, red if damaged	Viable if cells are elongated (Kogure et al., 1979) [35]	Not specified
**2. Arana** [21]	1. Sterile saline solution (0.9% NaCl, SS) 2. Seawater	1. Starvation in darkness or exposure to visible radiation. 2. Starvation + exposure to acid environment and to hydrogen peroxide H_2_O_2_	Not specified	1. Incubation on Tryptone Soy Agar (TSA) 2. Incubation on TSA + catalase (11 U/mL^−1^) for 24 h at 37 °C No culture	Not specified	LIVE/DEAD BacLight kit; Microscopy	Not specified	Flow cytometry + LIVE/DEAD BacLight kit
**3. Pinto** [19]	Deionized water + different concentrations of NaCl, depending on the strains	Temperature 4 °C without shaking	Several weeks	1. Incubation on TSA 2. Incubation on TSA + Sodium Pyruvate (SP) overnight at 37 °C No culture	Not specified	Not specified	Elongation method (Besnard et al., 2000) [36]	Not specified
**5. Dolezavola** [23]	Medium plates (M-FC agar base with rosolic acid)	Non-thermal atmospheric pressure plasma jet; Incubation at 37 °C	1 day	Culturability test not performed	Not specified	1. LIVE/DEAD BacLight kit; 2. ThioBarbituric Acid Reactive Substances assay (TBARS) method to measure lipid peroxidation products in cells	Not specified	Not specified
**6. Govers** [10]	LB broth	Heat treatment (high hydrostatic pressure)	15–18 h	Culturability test not performed	Not specified	Not specified	Not specified	Time-lapse fluorescence microscopy (TLFM)
**8. Chen** [2]	10 mL of sterile NaCl solution (0.9%)	Disinfection using chlorination and chloramination	30 min with chlorine; 2 h with chloramine	Culturability test not performed	CTC fluorescence intensity (respiratory activity); Raman microspectroscopy and spectral processing (metabolic activity)	LIVE/DEAD BacLight kit	Electron microscopy: bacteria became shorter	Flow cytometry
**9. Kim** [14]	LB cultures + 25 mL of fresh LB (1:1000)	Rifampicin during 30 min + 25 mL of LB with ampicillin (100 µg/ML^−1^); Incubation for 3 h	Overnight	No culture On LB agar plates after 16 h	Redox sensor green through flow cytometry (to assess the metabolic activity)	LIVE/DEAD BacLight kit	Transmission Electron Microscopy (TEM): spherical, empty cytosol;	Hemocytometer after staining with LIVE/DEAD BacLight kit
**10. Ogane** [25]	Sterile physiological saline (0.85%) solution	1. Sorbic acid + benzoic acid 2. Sodium hypochlorite NaClO; Incubation at 37 °C	30 days	Culturability test not performed	Damaged cells due to production of reactive oxygen species but technique not specified	LIVE/DEAD BacLight kit	Gram staining: small cocci	Flow cytometry and formula not specified
**11. Yamasaki** [6]	LB broth	Rifampicin Incubation at 37 °C	48 h	Culturability test not performed	Technique not speciified	Not specified	Not speciified	Not specified
**12. Wasfi** [26]	Deionized water	1. Starvation + low osmotic pressure 2. NaCl 0.9% 3. NaCl 0.9% + Ph 5 4. NaCl 4% + high osmotic pressure 5. NaCl 7% + high osmotic pressure. Incubation at 4 °C without shaking and in the dark	14 to 26 weeks	TSA every week No culture	Metabolic activity maintained by detection of expression of the 16S *rRNA* and *RpoS* genes by gel electrophoresis	Not specified	Transmission electron microscope (TEM): bacteria shorter than original strains	RNA expression via qRT-PCR
**13. Song** [11]	LB broth	Rifampicin (100 μg mL^−1^); Ampicillin	30 min for rifampicin; 3 h for ampicillin	Not specified	Not specified	Not specified	Not specified	Not specified
**14. Saima** [27]	Sterile distilled water	Not specified	33 weeks	Culturability test not performed	Not specified	Not specified	Not specified	Not specified
**15. Wilks** [28]	Artificial urine *	Urinary catheters: 1. Hydrogel latex + silver 2. Silicone	72 h	Not specified	LIVE/DEAD BacLight kit	Not specified	Not specified	Staining with SYTO 9 (total cell counts [TCC]), staining with propidium iodide (PI) (dead cells [dead]), and culture analysis (CFU counts) Calculation of VBNC using the following equation: $\%VBNC=\frac{\left[TCC-(CFU+dead)\right]}{TCC}$ **× 100**
**16. Zhu** [29]	LB broth	Chlorination + UV disinfection	Overnight	Heterotrophic plate counts (HPC); Incubation at 37 °C for 24 h No culture	CTC fluorescence	LIVE/DEAD BacLight kit	SEM; TEM	Flow cytometry
**17. Yadav** [30]	1. 2XYT (2x Yeast Extract Tryptone medium) broth for *E. coli*; 2. MHB (Mueller Hinton Broth) media for *K. pneumoniae*	Formic acid treatment	4 to 10 days	Checked by the standard agar plate method No culture	Metabolic activity: ATP production quantified by BacTiter-GloTM Microbial Cell Viability Assay; Respiratory activity: BacLight™ RedoxSensor™ CTC Vitality Kit	Propidium monoazide (PMA)	Not specified	BacLight™ RedoxSensor™ CTC Vitality Kit; Flow cytometry
**19. Chiang** [15]	PBS	UV and chlorine disinfection	Not specified	Agar plates No culture	DyeTox13 C-2 Azide-qPCR	Damaged membranes. LIVE/DEAD BacLight bacterial viability kit; Flow cytometry	Not specified	qPCR
**21. Li** [32]	LB broth	1. 20 µM CuSO_4_ for 12 h 2. Citric acid (pH 4.0) for 1 h 3. Cold shock at −20 °C for 1 h	16 h	Not specified	Suppressed DNA replication, reduced macromolecular synthesis, increased oxidative stress but techniques not specified	Damaged but techniques not specified	Not specified	Not specified
**22. Zhao** [33]	1. ASW (Artificial Sea Water) as control 2. ASW-YPD (Yeast Peptone Dextrose) 3. ASW-M9 (minimal medium) media	Anaerobically + low temperature (4 °C) 1. Low-nutrient environment	50 days	MacConkey agar plates No culture	LIVE/DEAD BacLight kit	Not specified	Not specified	qPCR and PMA-ddPCR
**24. Ni** [13]	Water samples (tap water and spring water)	Chlorine disinfectants	Not specified	Nutrient agar (NA) and R2A media using heterotrophic plate count Culturability test non specified	Metabolic activity maintained with potential infectivity (techniques not specified)	Intact membranes (techniques not specified)	Not specified	TaqMan-based qPCR (real-time fluorescence targeting 16S r-RNA gene)

* Peptone L37, yeast extract, lactic acid, citric acid, sodium bicarbonate, urea, uric acid, creatinine, sodium chloride, iron II sulfate, magnesium sulfate, sodium sulfate, potassium dihydrogen phosphate, di-potassium hydrogen, phosphate.

**Table 5:** Secondary outcomes—*Pseudomonas aeruginosa*.

Article Nb	Entry into VBNC State			VBNC State Confirmation (According to Pan’s Definition) Result/Method				VBNC Cells Quantification
	Culture Media	Growth Conditions	Duration Time	Culturability on Current Media	Metabolic/ Respiratory Activity	Membrane Integrity	Abnormal Morphology	Method
**4. Dwidjosiswojo** [22]	1. sterile water 2. Drinking water 3. Deionized water	3. Copper sulfate or copper nitrate + copper chloride	Not specified	Nutrient agar No culture	Metabolic activity maintained via FISH; Cytotoxic function reduced on CHO-9 (Chinese Hamster Ovary) cells with epifluorescence microscopy (staining DAPI)	Maintained (Bacterial viability kit Live/Dead Baclight)	Not specified	Not specified
**7. Dopp** [24]	20 mL deionized water	Copper sulfate (10 μM CuSO_4_); 20 °C; Darkness	24 h	Media not specified No culture	Not specified	Not specified	Not specified	DAPI staining + Fluorescence microscopy
**15. Wilks** [28]	Artificial urine *	Urinary catheter surfaces (1) Silicone (2) Latex hydrogel (3) a silver alloy-coated hydrogel latex	72 h	Not specified because the article is about biofilms structure	Not specified	Not specified	Not specified	Staining with SYTO 9 (total cell counts [TCC]), staining with propidium iodide (PI) (dead cells [dead]), and culture analysis (CFU counts) Calculation of VBNC using the following equation: $\%VBNC=\frac{\left[TCC-(CFU+dead)\right]}{TCC}$ **× 100**
**18. Hu** [31]	Not specified	Chlorine (0.1–1.0 mg/L)	1 h	Media not specified No culture	Reduced - Gas chromatography coupled with mass spectrometry - Raman spectroscopy	Not specified	Not specified	Not specified
**19. Chiang** [15]	PBS (poor media)	1- UV 2- Sodium hypochlorite	1- 30 and 60 min 2- 1 h	Agar plate LB Culturability not specified	Reduced. DyeTox13-qPCR to measure enzymatic activity	Maintained Live/Dead Baclight Flow cytometry	Not specified	PMA-qPCR
**20. Guo** [3]	Not specified	Chlore	7 days	Media not specified No culture	Not specified	Maintained Flow cytometry	Not specified	The number of VBNC bacteria was calculated by subtracting the number of viable cells from the number of cultivable cells (formula not specified)
**23. Fan** [34]	Not specified	1. UV, 2. NaClO 3. PAA (Paracetic Acid)	30 min	Media not specified 1. No culture 2. No culture 3. Residual growth	Maintained ATP production (BacTiter Glo microbial cell viability assay)	Not specified	Not specified	Calculating the disparity between the viable cell count established by the PMA-qPCR method and the count of culturable cells determined by the HPC method: **VBNC cells count = viable cells count − culturable cells count**
**24. Ni** [13]	Source water Potable water Tap water	Chlorine disinfectants	Not specified	Media not specified No culture	Maintained Not specified	Intact Method non specified	Not specified	Taqman based qPCR (real time fluorescens targeting 16s rRNA)

* Peptone L37, yeast extract, lactic acid, citric acid, sodium bicarbonate, urea, uric acid, creatinine, sodium chloride, iron II sulfate, magnesium sulfate, sodium sulfate, potassium dihydrogen phosphate, di-potassium hydrogen, phosphate.

**Table 6:** Synthesis of operating conditions enabling the study of VBNC bacteria specific to each bacterial group or common to both groups.

Enterobacteriaceae		*Pseudomonas aeruginosa*	
VBNC State Induction			
**Nutrient depletion** *	Oligotrophic medium (Minimal medium or Yeast-Peptone-Dextrose broth) Relatively rich medium (Luria Bertani broth, Muller Hinton broth) NaCl solutions NaCl solution + oligotrophic medium		
	Phosphate Buffered Saline Drinking Water: tap or spring Nutrient free water: distilled or desionized		
**Additional specific growth conditions**	4 °C Visible radiations Heat Acids (sorbic, benzoic, formic) H_2_O_2_ Antibiotics High osmotic pressure		20 °C Cooper (sulfate or nitrate or chloride) Peracetic acid
	Darkness UV Chlorine		
**VBNC state confirmation**			
**No culture (or poor culture)**	Current agar media		
**Respiratory activity maintained**	CTC staining		FISH DAPI staining
**Residual metabolic activity maintained**	Redox sensor green + flow cytometry Gene expression (electrophorese)		Gas chromatography + mass spectroscopy
	DyeTox13 Green C-2 Azide + qPCR Raman spectroscopy ATP production		
**Membrane integrity maintained**	ThioBarbituric Acid Reactive Substances assay Propidium monoazide (PMA)		
	Live/Dead Baclight staining		
**Abnormal morphology**	Scanning electron microscopy Transmission electron microscopy		
**VBNC quantification**			
	flow cytometry + Live/dead or Redox Sensor or CTC Time-lapse fluorescence microscopy (TLFM) qRT-PCR		flow cytometry + Live/dead or DAPI
	PMA-ddPCR/qPCR TaqMan-based qPCR		
**VBNC Resuscitation**			
**Culture media** **	Medium Broth Glucose Yeast Extract (MBGYE) M9 broth Phosphate Buffered Saline TS Broth Yeast Peptone Dextrose (YPD)		Deionized water
	Agarose gel pads Luria Bertani broth		
**Additive**	Sodium pyruvate/thioglycolate Catalase Growth culture supernatant Trace mineral supplement Tween 20 + diluted BHI (1/8) Amino-acids disinfectant neutralizer: Sodium thiosulfate		Cooper chelator: Sodium diethyldithiocarbamate trihydrate (DDTC) + cooper sulfate
**Growth conditions**	Incubation at optimal growth temperature		

* For Enterobacteriaceae: with at least 1 additional specific growth condition; For *Pseudomonas aeruginosa*: with or without specific growth condition. ** For Enterobacteriaceae: with or without 1 additive; For *Pseudomonas aeruginosa*: no additive except if cooper was used for VBNC state induction.

## Data Availability

No new data were created or analyzed in this study. Data sharing is not applicable to this article.
